# Sport and sexual recovery after total hip arthroplasty in young adults: a retrospective cohort study

**DOI:** 10.1007/s00402-024-05544-7

**Published:** 2024-09-23

**Authors:** Alberto Di Martino, Chiara Di Censo, Matteo Brunello, Valentino Rossomando, Claudio D’Agostino, Giuseppe Geraci, Francesco Traina, Cesare Faldini

**Affiliations:** 1 1st Department of Orthopaedics and Traumatology, IRCCS - Istituto Ortopedico Rizzoli, Via Giulio Cesare Pupilli 1, Bologna, 40136 Italy; 2 Department of Biomedical and Neuromotor Science-DIBINEM, University of Bologna, Bologna, 40136 Italy; 3 Department of Ortopedia-Traumatologia e Chirurgia Protesica e dei Reimpianti di Anca e Ginocchio, IRCCS Istituto Ortopedico Rizzoli, Bologna, 40136 Italy

**Keywords:** Sport activity, THA, THR, Quality of life, Quality of sexual activity SQoL

## Abstract

**Introduction:**

: Total Hip Arthroplasty (THA) is the main treatment for end-stage degenerative hip arthrosis in the elderly, while became increasingly performed as treatment of secondary arthrosis in younger patients, a population at high functional requests in terms of resumption of physical activity and resumption of sexual activity. This study evaluates the physical, sports recovery and the sexual quality of life in young patients undergoing primary THA.

**Materials and methods:**

Patients undergoing primary THA aged between 18 and 45, operated in a 10 year timeframe, were selected. Demographic, clinical, and radiological data were collected. The Harris Hip Score (HHS) was assessed before and after the surgery. The UCLA Activity Score was collected, sport participation in pre and post-operative period was acquired. The quality of sexual activity (SQoL) before and after surgery was analysed through a qualitative questionnaire. Collected data were also compared on the surgical approach, namely Direct Anterior (DAA), Postero-Lateral (PL) and Direct lateral (DL).

**Results:**

The population consisted of 242 THA implanted in 232 patients, including 143 males and 89 females, with an average age of 37.4. The mean follow-up period was 51.0 months, with a minimum of 2.9 months and a maximum of 122.6 months. Postoperatively, the average HHS was 90.29 ± 0.9 points, compared to 62.43 ± 1.34 points preoperatively (*p* < 0.001). The UCLA Activity Score in the postoperative period was 7.17 ± 0.17 matching to intermediate impact sport activity. The 64% of patients reported an improvement in SQoL after-surgery, 73% of which being females that show a significant improvement in SQoL compared to males (*p* = 0.046). By surgical approach comparison, DAA patients demonstrated better UCLA Activity Score (*p* = 0.037) and Return to sport (*p* = 0.027) compared to PL and DL.

**Conclusion:**

Primary THA surgery in young adults can improve the level of physical activity and promote the involvement of subjects in moderate impact sports. Patients showed a better quality of sexual life compared to the preoperative period, an effect more evident in female patients.

## Introduction

Total Hip Arthroplasty (THA) is regarded as one of the most successful procedures in orthopaedics because of its efficacy in functional limitations and joint pain associated with degenerative hip diseases [1]. The main indication for THA is primary OA [2, 3]. This procedure has traditionally been performed in the elderly, typically after the age of 65, taking into consideration that implant survival could reach 20 years in most patients [4]. With the improvement in biomaterials technology and biocompatibility, and with the implementation of surgical techniques that promote faster recovery and minimally invasive procedures, the survival of implants has increased, and therefore the indications to THA surgery have been extended including younger patients. The most common diseases whose sequelae may require THA before the age of 45 include developmental dysplasia of the hip, Slipped Capital Femoral Epiphysis (SCFE), Legg-Calvé-Perthes disease, avascular necrosis of the hip, and traumas; these conditions may lead the patients to disability and to a reduced quality of life which begins already in late adolescence and early adulthood.

The resumption of physical activities (RoPA) and sports activities (Return to Sport, RtS), along with the restoration of sexual quality of life (SQoL) [5] shows unique needs to be considered in the postoperative outcome of THA in a young and active population. These indicators have an impact on physical and psychological health and are adversely affected by hip diseases in patients below 45 years-old [6]. However, these aspects are commonly underestimated and considered complementary in the assessment of the outcomes of THA surgery and implant performance in young adult patients [7, 8]. On the other hand, this category is particularly sensible to the long-term survival of the implants, and to the chances to keep or improve their lifestyle [9]. Notably, Wright and Young [10] claimed that RtPA was the third most important expectation of patients after THA, highlighting the importance of a correct and precise patient education by the surgeon on the topic. It is known that patients are able to satisfactorily perform a sport discipline after THA, and more frequently surgeons suggest an active lifestyle in the postoperative period [5]. This underlines that providing patients with accurate information regarding postoperative recovery and proper implant management are fundamental topics during outpatient consultations.

Return to sexual activity in patients with THA is a crucial issue more specific for younger patients [11], but this subject is underdiscussed in literature. Wall et al. [12] reported that patients would receive more information about the chance of sexual practice after THA implantation, knowing that sexual activity may expose the hip to potentially unsafe positions, posing a risk of complications such as dislocation of the implant [13]. 

Investigating the safety of sport and sexual activity in young patients with THA is therefore crucial to ensure proper discussions with the patient in the outpatient setting. Commonly, these aspects are under-discussed, but a correct information about an appropriate resumption of sport or sexual activity may be beneficial for patients during postoperative recovery [14].

Therefore, the aims of the current study are: (1) to verify whether THA in patients below 45 years-old allows an adequate restoration of physical activity and a return to sports; (2) to verify the existence of an improvement in the SQoL in patients of both genders after THA surgery; (3) to analyse whether the surgical approach (DAA, PLA or DLA) determines different results in term of RoPA, RtS or SQoL.

## Materials and methods

Patients undergoing primary THA before the age of 45, performed between January 1st, 2012 and December 31st, 2022 at the Authors’ Institution, were enrolled in the study. This study was approved by Authors Institute Ethics Committee (approval no. CE-AVEC 362/2023/Oss/IOR). The inclusion criteria were (a) to be subjected to primary THA surgery, (b) age at surgery between 18 and 45 years. The exclusion criteria were (a) history of oncologic disease or presence of bone metastases (b) traumatic fracture of the hip (c) previous infectious diseases (d) patients with previous THA surgery (e) patients operated on for hip resurfacing. In the study period, 242 THAs were implanted in 232 patients. The single operated hip was considered as a statistical unit. The demographic, clinical and radiological data were collected from the clinical records of the internal hospital database. Particularly, the following clinical information were taken into account: diagnosis, age at the surgery, duration of hospitalization (calculated in days), American Society of Anaesthesiologists (ASA) scores, pain at the second postoperative day considering the Numerical Rating Scale (NRS), requirement and number of transfusions, surgical time (considered by the time from incision to end of suture), surgical approach (DAA, DL or PL). Intra and post-operative complications were recorded. Patients were followed at 1, 3, 6 month, one year, and then yearly. The RoPA, RtS and SQoL were assessed at 6 months post THA surgery by the administration of questionnaires. The Harris Hip Score was used for assessment of overall patients function in the Italian validated version [15]; HHS [15, 16] it is a multi-disability measurement tool that allows the assessment of the Activities of Daily Living (ADL) and the functionality of patients with hip pathology. The RoPA was investigated by the University of California at Los Angeles Score [17, 18]. It is made up of a 10-level scale. RtS was evaluated investigating the type of sport activity performed by the patient in the pre and postoperative period. Patients’ behaviour was analysed postoperatively and it was compared to the preoperative activity, in terms of continuation and interruption of their previous sport practice, or new engagement postoperatively. The level of impact of the practiced sport discipline was collected and classified as “low”, “intermediate” and “high” impact loading on the joint after THA [19, 20] (Table 1). The SQoL reported by the patient in the pre- and post-operative periods was investigated using closed-ended structured questionnaire, exploring if the patient considers the quality of the intercourses to be improved or worsened post-operatively, or if no changes were experienced in the quality of sexual activity because of limitations at the operated hip. The SQoL was analysed on the whole population and according to the patient’s gender.

**Table 1:** Sport disciplines grouped for level of impact on hip joint

Level of impact of practiced sports	
Low	Walking Cycling Swimming Ballroom dancing Golf Water aerobics
Intermediate	Horseback riding Free weight lifting Rock climbing Low-impact aerobics Tennis In-line skating
High	Soccer Basket Volleyball Football Running/Jogging

Collected data were also analysed with respect to the surgical approach used during THA surgery: Direct anterior (DA), direct lateral (DL) and posterolateral (PL) approaches were considered.

### Radiographic evaluation

The radiological evaluations were conducted by examining standard postoperative radiographs in antero-posterior view. Cup inclination and stem alignment were evaluated on the coronal plane. Cup inclination was measured as the angle formed between the line passing through the edge of the acetabular cup and a horizontal reference line tangent to the ischiopubic tuberosities. To assess the correct placement of the acetabular component, reference was made to the Lewinnek safety zone, (40° ± 10°) [21]. The alignment of the stem is the angle formed by the diaphyseal axis of the femur and the longitudinal axis of the prosthetic stem on AP radiograms. This parameter is an index of varus or valgus of the implant, considered optimal in a range of 0° ± 5°: a greater angle is considered varus, while the smaller angle determines a valgus positioning [22].

### Statistical analysis

Patients eligible for the study were divided into three groups based on the surgical approach, and the data relating to the resulting populations were compared using the ANOVA and Chi-square tests SPSS v.14.0 software (SPSS Inc., IBM, Chicago, IL, USA) and statistical test results were considered significant with a p-value < 0.05.

## Results

The study population consisted of 242 THAs implanted in 232 patients (89 women and 143 men) with an average age of 37.4 years (range 18–45 years). The average length of in-hospital stay was 7 days (range 3–35 days). The mean follow-up period was 51.0 months, with a minimum of 2.9 months and a maximum of 122.6 months. The indications to THA surgery were developmental dysplasia of the hip (*n* = 69), avascular necrosis of the femoral head (*n* = 67), post-traumatic hip arthritis (*n* = 28), secondary hip arthritis not otherwise specified (*n* = 22), sequelae of Legg-Calvé-Perthes disease and Slipped Capital Femural Epiphysis (*n* = 13), while the remaining 43 patients were generically grouped in “others” specified secondary arthritis of the hip. As regards the ASA score, 51% (123/242) of patients fell into ASA I, 44% (107/242) into class II and 5% in class III (12/242). 154 (63.6%) surgeries were performed by DAA, 50 (20.6%) by PL approach and 38 (15.7%) by DL. Duration of surgery was on average 99.27 ± 5.18 min. Transfusions were necessary in an overall 26% (63/242) of patients, which required an average of 2 red blood cell packs per patient in the post-operative period. Analysis of medical records showed that 25% of patients (61/242) reported a NRS pain score of 0 at the second postoperative day, while for the remaining 75% (181/242), an average score of 2.1 ± 0.3 was recorded. During follow up, 11 patients required surgical revision of the implant because of infection (*n* = 3, managed by 2-stage component replacement), while 8 developed mechanical complications (dislocation, *n* = 3; painful joint, *n* = 3; cup malpositioning, *n* = 2). Study population and its characteristics are summarized in Table 2.

**Table 2:** Cohort composition. SCFE: slipped capital femoral epiphysis NRS: Numerical Rating Scale

Cohort composition	
**Total number of THAs**	242
**Total number of patients**	232
**Gender**	89 women, 143 men
**Average age (years)**	37.4 (range 18–45)
**Average length of hospital stay (days)**	7 (range 3–35)
**Indications for THA**	
- Developmental dysplasia of the hip	69
- Avascular necrosis of the femoral head	67
- Post-traumatic hip arthritis	28
- Secondary hip arthritis not otherwise specified	22
- Sequelae of Legg-Calvé-Perthes disease and SCFE	13
- Other specified secondary arthritis of the hip	43
**ASA score**	
- Class I	51% (123/242)
- Class II	44% (107/242)
- Class III	5% (12/242)
**Surgical approaches**	
- DAA (Direct Anterior Approach)	154 (63.6%)
- PL (Postero-Lateral Approach)	50 (20.6%)
- DL (Direct Lateral Approach)	38 (15.7%)
**Average duration of surgery (minutes)**	99.27 ± 5.18
**Transfusions needed**	26% (63/242)
**Average number of blood packs per patient**	2
**NRS pain score on the second postoperative day**	
- NRS 0	25% (61/242)
- Average NRS 2.1 ± 0.3	75% (181/242)
**Mean Follow-up (month)**	51,0 (21,0–75,0)
**Complications during follow-up**	
- Surgical revision for infection	3
- Mechanical complications:	8
- Dislocation	3
- Painful joint	3
- Cup malpositioning	2

### Return to function and sport

On average, patients showed preoperative HHS scores of 62.43 ± 1.34 points, that significantly improved after surgery to an average of 90.29 ± 0.9 points (*p* < 0.001). The RtPA, assessed by UCLA score, resulted in average 7.17 ± 0.17 points, corresponding to regular participation in low impact activities such as cycling, swimming or gentle jogging.

**Fig. 1:**
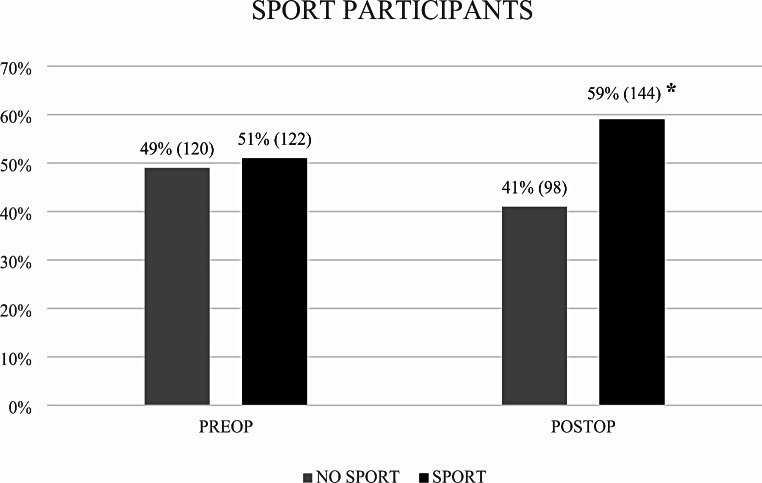
Sport participation before and after the surgery, (* statistical difference)

Overall, the rate of participants to any sport significantly increased after surgery (*p* = 0.045) (Fig. 1). The 49% (120/242) of patients were engaged in sport activities before surgery, while 59% (144/242) were involved in sports at the 6-month postoperative follow-up.

Overall, 40% (97/242) of patients kept the sports activity that was carried out before the operation even in the post-operative period. 19% (46/242) started a sport activity after surgery, while 9% (22/242) stopped the activity that was practiced preoperatively; 32% (77/242) of patients did not practice any sport before and after surgery (Table 3).

**Table 3:** Rate of participants to sport after THA surgery

Return to sport after surgery	No (%)
No sport participation	77 (32%)
Started sport after surgery	46 (19%)
Avoided sport after surgery	22 (9%)
Continue sport after surgery	97 (40%)
More than one sport preoperatively	9 (4%)
More than one sport postoperatively	11 (5%)

**Fig. 2:**
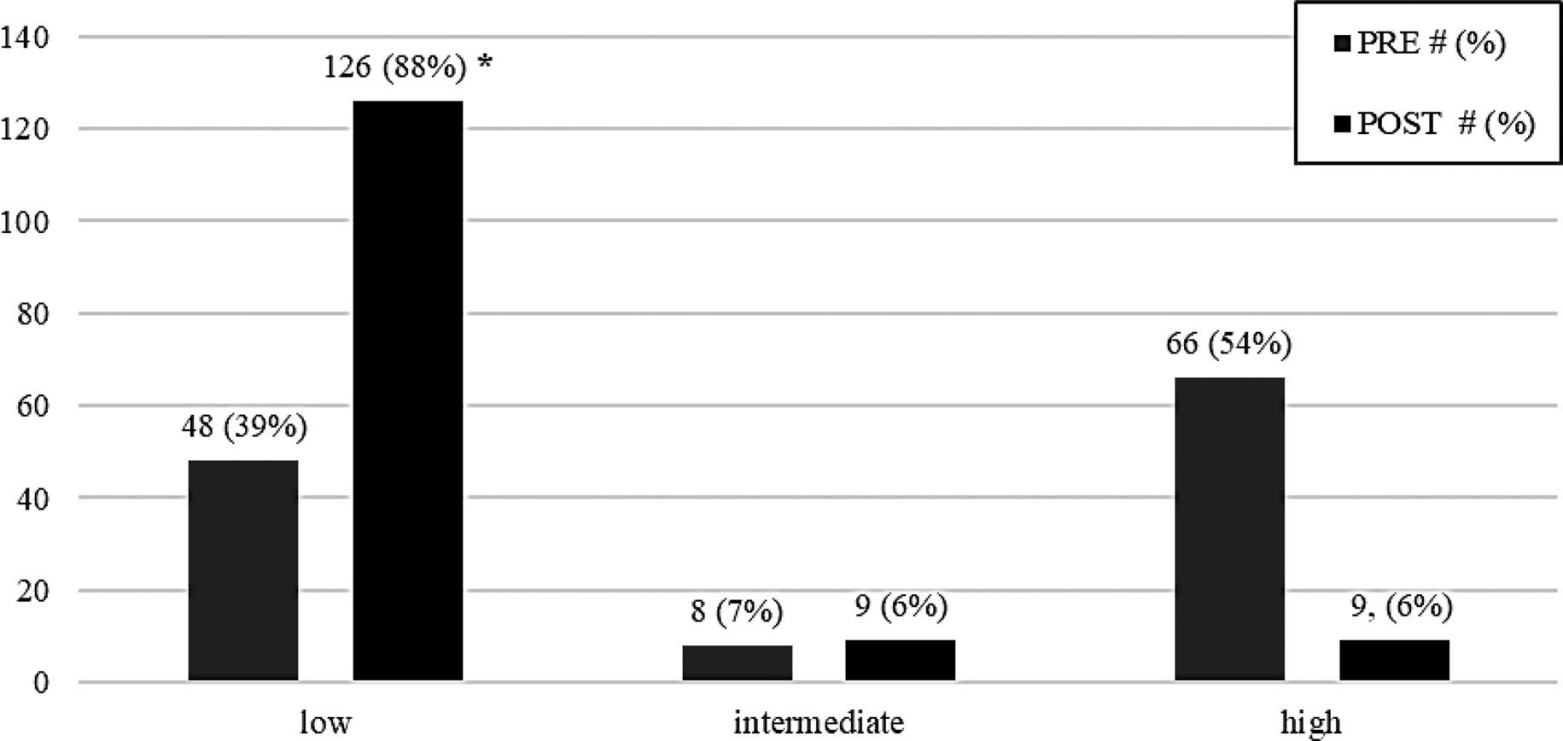
Sport participation differentiated by impact before and after the surgery, (* statistical difference)

The most practiced sports disciplines included trekking, gym workouts, and swimming. Nine patients (4%) were engaged in more than one sport preoperatively, while in the postoperative period the number of patients playing more than one sport was 11 (5%). A decreased participation to high impact loading sports, like running or soccer [19], was recorded in the postoperative period (Fig. 2); conversely, a significant increase in low impact sports was registered in the postoperative period with respect to the preoperative (*p* < 0.001).

### Sexual quality of life

The 64% (*n* = 149) of patients reported an improvement in the quality of sexual intercourse after surgery, while 34% (*n* = 79) of patients noted no difference; 2% (*n* = 4) of patients referred a worsening in SQoL after surgery (Table 4). When gender differences were considered, significantly more females reported an improvement in postoperative SQoL compared to males (*p* = 0.046). The 73% (*n* = 65) of female patients reported improved of quality of sexual intercourse post-operatively, 24% (*n* = 22) had unchanged quality of sexual relationship, and 2% (*n* = 2) showed a worsening. On the other hand, the male population presented 59% (*n* = 83) of patients reporting an improvement after surgery, 40% (*n* = 58) of cases had no difference, and 1% (*n* = 2) showed a worsening; one of these two patients reported a two staged surgical treatment due to required component infection in anamnesis.

**Table 4:** Results of sex quality of Life evaluated by a three

	Total *n*, (%)	Females *n*, (%)	Males *n*, (%)	
Worsened	4 (2%)	2 (1.5%)	2 (1%)	NS
Unchanged	79 (34%)	22 (24%)	58 (40%)	NS
Improved	149 (64%)	65(73%)	83 (59%)	*P* = 0.046

### Subgroup analysis according to surgical approach

In the DA group, the number of female patients was 42.8% (*n* = 66), while 57.2% (*n* = 88) were males; in the DL group, there were 44.7% (*n* = 17) women and 55.3% (*n* = 21) men; in the PL group there were 22.2% (*n* = 11) women vs. 77.8% (*n* = 39) men. A significantly higher number (*p* = 0.036) of females was recorded in PLA group respect with the DAA and DLA groups. No differences in terms of average age at surgery (*p* = 0.085), days of in-hospital stay (*p* = 0.282), number of revisions (*p* = 0.067) and infections (*p* = 0.592) was observed comparing groups. No significant differences were found on the rate of complications (*p* = 0.067): in the DAA group, 5/154 (3.2%) patients underwent a surgical revision, in 2 cases for periprosthetic joint infection managed by Debridement, Antibiotic and Implant Retention (DAIR) in one case, and by full implant revision in another. The remaining 3 patients had a dislocation of the femoral component managed by revision THA. In the PL group, there was one surgical revision due to pain and a snapping sensation; another patient reported an intraoperative fracture managed by metal wiring. In the DL group there were 5 surgical revisions: in one case for septic loosening of the implant managed by explant and surgical debridement, with staged reimplantation. In another patient, wear of the polyethylene component occurred one year after surgery, which was managed by revision of the acetabular component. The same patient reported a dislocation of the implant 4 months after acetabular revision, which was managed by total revision of the implant. One patient underwent explant and reimplantation of the prosthesis in the immediate post-operative period for cup malposition. These results are summarized in Table 5.

**Table 5:** Patient demographics and clinical characteristics according to the surgical approach

	DAA	DLA	PLA	
THA number(%)	154 (63.6)	38 (15.7)	50 (20.6)	
Females (%)	66 (42.8)	17 (44.7)	11 (22.2)	*p* = 0.036
Males (%)	88 (57.2)	21 (55.3)	39 (77.8)	chi-square
Age at surgery (range)	37.6 (20–46)	35.8 (18–46)	39.2 (20–48)	*p* = 0.085 ANOVA
Time of in-hospital stay (range)	7.5 (3–35)	8.4 (4–15)	7.7 (4–13)	*p* = 0.282 ANOVA
Revision surgery (%)	5/154(3.2)	5/38(12.2)	1/50(2)	*p* = 0.067 chi-square

**Fig. 3:**
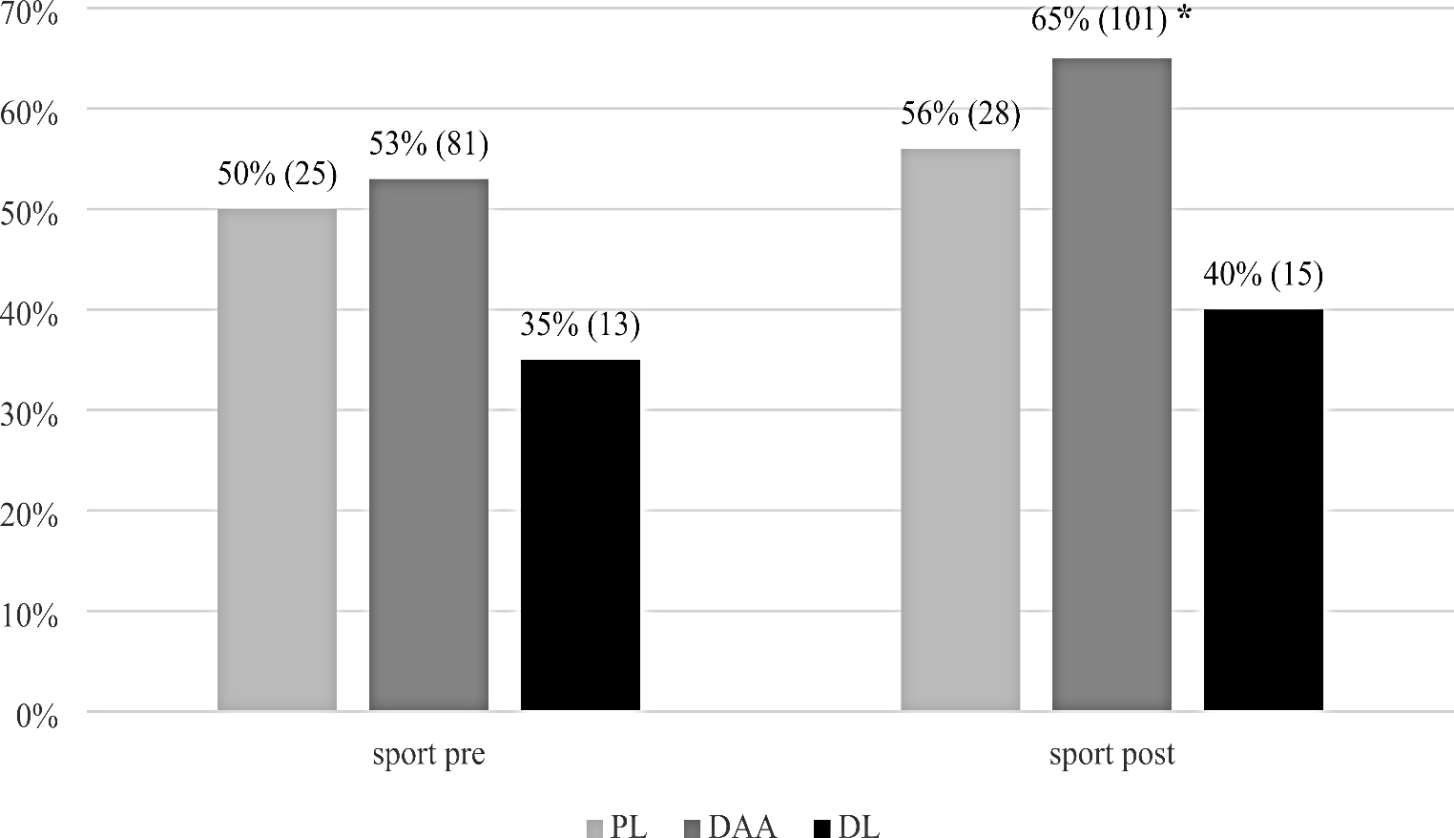
Sport participation [%, (no)] grouped for the different surgical approaches in the preoperative and postoperative period

No differences were observed comparing patients divided by surgical approach in terms of postoperative HHS (*p* = 0.992). Patients that underwent DAA THA showed a return to a significantly higher level of physical activity compared to other surgical approaches, as assessed by UCLA score questionnaires (*p* = 0.037). In particular, in DAA patients, a greater result with an average of 7.2 (range 3–10) points of UCLA Activity Score was achieved, compared to 6.8 (range 1–9) for the DL and 6.9 (range 5–9) for the PL (*p* = 0.037). Evaluating the rate of return to sport or abandonment (Fig. 3), in the post operative period 65% (101/154) of patient with DAA THA were engaged in a sport discipline, while 40% (15/38) with DL THA and 56% (28/50) with PL THA practiced sport after surgery. No significant differences were highlighted comparing the RtS in the PL and DL approach groups, while DAA patients had significantly more sport participants postoperatively compared to preoperative (*p* = 0.027) (Fig. 3); interestingly, DAA patients had significantly less patients engaged in sports preoperatively compared to PL and DL (*p* = 0.042). Patients operated on for DAA showed significantly better SQoL compared to those patients operated through other surgical approaches (*p* = 0.009).

### Radiographic evaluation

The average cup inclination in the entire patient cohort was 39.78 ± 1.51°, while stem alignment averaged − 0.05 ± 0.49°. Comparing the set of angles of cup inclination with respect to the safety zone defined by Lewinnek, this was respected in 66% of patients, and 34% were outside the range. No significant differences were reported in terms of cup inclination (*p* = 0.067) and stem alignment (*p* = 0.175) among the three surgical approaches. In particular, as regards cup positioning, 67.5% (104/154) of the cases in the DA group, 81.6% (31/38) of the DL and 58% (29/50) of the PL group were within the range. Regarding stem alignment, 85.8% (97/154) of cases in the DA group, 73.3% (28/38) in the DL group and 70% (35/50) in the PL group presented correct alignment. Data are summarized in Table 6.

**Table 6:** Radiographic parameters per group analysis

	Direct anterior	Direct Lateral		Posterolateral	
Cup positioning Safe zone (%) Out of safe zone (%)	104 (67.5) 50 (32.5)		31 (81.6) 7 (18.4)	29 (58) 21 (42)	*p* = 0.067 chi-square
Stem alignment In range (%) Out of range (%)	97 (85.8) 16 (14.2)		28 (73.3) 10 (26.3)	35 (70) 15 (30)	*p* = 0.175 chi-square

## Discussion

The present study shows that in a cohort of patients undergoing THA surgery before the age of 45, physical activity, return to sport and sexual quality of life underwent a significant improvement in the postoperative period compared to the pre-operative. Young adult patients undergoing THA were in most cases able to engage in low-impact physical activities, and they showed an elevated resumption of level of sports activity already six months after surgery. THA surgery led to a significant improvement in SQoL in patients’ cohort, with a higher improvement observed in females compared to males.

The diseases leading to secondary degenerative arthritis mirror those reported by the Regional Registry of Implants of Emilia Romagna Region (R.I.P.O.) [23], which includes sequelae of Developmental Dysplasia of the hip, Osteonecrosis of the femoral head, and post-traumatic degenerative arthritis of the hip as the three most common causes in descending order. Collected clinical data in terms of American Society of Anaesthesiologist class (ASA Score), pain on the second postoperative day and the transfusion requirements were in line with current literature on THA in young adults [24, 25]. As expected, a significant improvement in HHS scores were observed post-operatively compared to the pre-operative period (90.29 ± 0.9 vs. 62.43 ± 1.34; *p* < 0.001), suggesting an improvement in hip joint function and pain relief, independently from the surgical approach that was used.

A significant improvement in terms of RtPA was observed postoperatively. Magan et al. [26] conducted a meta-analysis that analyzed 2297 patients of all ages (93,9%; 95% CI, 82,2%-99,5%; *p* < 0.001) from eleven clinical studies, and found that they could return to practice a generic physical activity 6 to 12 months after surgery in 93.9%% comparable in our cohort in which 92% of patients was able to score 6 or more points at the UCLA Activity Score.

Enrollment in a sport discipline is not the only goal of young patients undergoing THA, which can be also interested in gaining a certain level of performance. Malcol et al. [27] in a clinical trial over 228 patient grouped per general and under 45 patients, found that THA patients under the age of 30 perform physical activity at a comparable level with respect to coetaneous without hip diseases in terms of UCLA score, confirming the optimal physical recovery of THA patients, similar to subjects with healthy hips. Our study underlines an increase in the overall number of sport practitioners after the surgery was observed, with 19% of patients not engaged in sports in the preoperative period which started a sport discipline after THA. Ortmaier et al. [9] analysed a population without age limitation, and observed satisfactory results in terms of RtS. Furthermore, they compared the sport disciplines performed by patients pre- and post-operatively, and observed a preference for low-impact activities including trekking, swimming or cycling. These data are in line with our analysis, where we observed an UCLA Activity score of 7.17 ± 0.17 corresponding to a moderate impact physical activity. These findings support the engagement in sports for patients operated on THA [28]. The 59% of patients in our cohort were able to practice sports at a 6 months from surgery. The sport disciplines more practiced were swimming, gym and cycling, all considered as low impact activities [29]. According to Streck et al. [28] the majority of patients undergoing THA are capable of engaging in low impact sports two years after-surgery without an increase in the risk of adverse events, including failure and revision surgery. Moreover, practicing a high activity level sport after primary THA does not shorten the survivorship of the implant at a short to mid-term follow up. Van der Weegen [30] claims that a less restrictive lifestyle following THA does not result in higher dislocation rates; moreover, an early and more successful RtS is associated to increased patient satisfaction.

We observed a significant reduction in high impact sport activities after THA, and an overall increase in low impact sports practice. The sport disciplines have been classified into three categories, “low”, intermediate and high impact sport, according to Clifford et al. [19] that list the several sport disciplines according to the loading impact on the prosthetic implant. Several evidences [9, 31, 32] in literature suggest the preferential adhesion to low impact sport disciplines after THA.

Our analysis indicates a better RtPA outcomes in THA implanted through the minimally invasive DAA compared to PL or DL approaches (*p* = 0.037). We also found that the UCLA score was higher in DAA THA (UCLA score 7.2) compared with patients operated on by other approaches. Although the relationship between physical and sports activity outcomes and surgical approach has been explored only partially in literature, our study supports how DAA yields better results compared to PL and DL approaches. According to Martusiewicz [33], patients with DAA THA experience shorter recovery times, reduced dependence on crutches, and a quicker return to daily activities compared to patients undergoing PL THA. DAA is well-known for being a muscle sparing surgical approach. It is associated to known benefits in terms of bleeding, early return to function and risk of dislocation [15, 34–37]. In particular, it is associated with a shorter hospitalization stay because of better recovery in the early postoperative period [38]. Improved function in the early postoperative rehabilitation observed in DAA THA may be one of the determinants of RtPA observed at 6 months after surgery.

Return to sexual activity in patients with THA, unlike RtS and RtPA, is an issue specific for young patients [11]. Our findings indicate that 64% of patients perceived an improvement in their Sexual Quality of Life (SQoL) postoperatively compared to the preoperative. When gender-related data were analyzed, a greater proportion of women reported an increase in SQoL following THA compared to men (71% vs. 60%). Despite the good results, the topic is underdiscussed in literature, and the information provided to patients in this regard are usually insufficient [11]. Accurate patients’ information regarding the resumption of sexual activity after THA is crucial to reduce the risk of instability and dislocation associated with the specific limb positions assumed during sexual intercourse [14]. However, most surgeons do not sufficiently discuss the topic with the patients in the pre- and post-operative period [14], this occurred for several reasons, including sensibility to the issue and embarrassment. Evidence suggests that patients would benefit from being informed about possible issues regarding sexual activity [39].

The patients should receive adequate and tailored information based on age-related, as well as socio-cultural and gender-specific aspects. Therefore, a thorough clinical interview is necessary, which takes into account these aspects and properly educates the patient based on not only clinical-functional characteristics but also gender-specific ones [40, 41].

In the review by Neonakis et al. [42], encompassing 16 articles and a cohort of 2391 patients, a positive impact of THA on SQoL was reported, mainly in the female population, in line with our results that shows 71% female patients reported an enhanced QoL with respect to 60% of males.

Most patients are able to resume satisfactory sexual activity without limitations approximately three months after the surgery. Current recommendations state that one and three months after THA, sexual activity is allowed but with restrictions on the positions assumed during intercourse, avoiding the extreme flexion and adduction, and extension with internal rotation. Many authors reported that the supine position, with the hips abducted and externally rotated, is the safest for both genders, but it is particularly favoured by females. Charbonnier et al. [13] studied with motion capture technology and Magnetic Resonance Images the movements at risk during sexual intercourse on two volunteers with hip prostheses of different sexes. They found that the female population was prone to higher degrees of flexion, abduction, and external rotation compared to males, leading to a greater risk of impingement. In most cases, excessive flexion (> 95°) was associated with a risk of posterior instability.

The current study has several limitations. The collection of data regarding the practiced sporting activity, the HHS and UCLA scores were conducted through telephone interviews between December 2022 and February 2023, so there is a temporal selection bias regarding the collection of information on the database created from January 2012 to December 2022. Furthermore, the structured closed-ended questionnaire design constructed for SQoL did not consider physiological, personal, and sociocultural factors associated with sexual activities; finally, parameters that quantified sexual function in terms of frequency and duration were not considered.

In conclusion, after THA surgery, young patients below 45 years are able to return to sport, usually at the same level they performed before surgery; interestingly, a significant group of patients decided to engage in sport activity after surgery, witnessing the return to function and the decrease of pain. The return to sexual activity after surgery is noticeable, even if it remains an underexplored topic during the acquisition of the informed consent, and that together with the return to sport, is a major determinant of the overall quality of life in young and active adult patients. The implementation of minimally invasive approaches, like DAA, may promote sport activity and return to function, speeding up the recovery of the patients. In conclusion, at present THA in the young active adults should not be procrastinated if joint degeneration compromises the overall quality of life and function; the results of the current study may improve doctor-patients communication, tailor expectations and help defining predictable outcomes in this peculiar patients’ population.
